# Trajectories of Adaptive Behaviors During Childhood in Females and Males in the General Population

**DOI:** 10.3389/fpsyt.2022.817383

**Published:** 2022-03-23

**Authors:** Tomoko Nishimura, Takeo Kato, Akemi Okumura, Taeko Harada, Toshiki Iwabuchi, Md. Shafiur Rahman, Tomoya Hirota, Michio Takahashi, Masaki Adachi, Hitoshi Kuwabara, Shu Takagai, Yoko Nomura, Nagahide Takahashi, Atsushi Senju, Kenji J. Tsuchiya

**Affiliations:** ^1^Research Center for Child Mental Development, Hamamatsu University School of Medicine, Hamamatsu, Japan; ^2^United Graduate School of Child Development, Hamamatsu University School of Medicine, Hamamatsu, Japan; ^3^Department of Psychiatry and Behavioral Sciences, Weill Institute for Neurosciences, University of California, San Francisco, San Francisco, CA, United States; ^4^Department of Clinical Psychological Science, Graduate School of Health Sciences, Hirosaki University, Hirosaki, Japan; ^5^Faculty of Medicine, Saitama Medical University, Saitama, Japan; ^6^Department of Child and Adolescent Psychiatry, Hamamatsu University School of Medicine, Hamamatsu, Japan; ^7^Queens College and Graduate Center, City University of New York, New York, NY, United States; ^8^Department of Child and Adolescent Psychiatry, Nagoya University Graduate School of Medicine, Nagoya, Japan

**Keywords:** trajectory, adaptive behavior, sex differences, neurodevelopmental traits, childhood, autism spectrum disorder, attention deficit hyperactivity disorder, cognitive ability

## Abstract

Little is known about the trajectory patterns and sex differences in adaptive behaviors in the general population. We examined the trajectory classes of adaptive behaviors using a representative sample and examined whether the class structure and trajectory patterns differed between females and males. We further explored sex differences in neurodevelopmental traits in each latent class. Participants (*n* = 994) were children in the Hamamatsu Birth Cohort for Mothers and Children (HBC Study)—a prospective birth cohort study. Adaptive behaviors in each domain of communication, daily living skills, and socialization were evaluated at five time points when participants were 2.7, 3.5, 4.5, 6, and 9 years old using the Vineland Adaptive Behavior Scales–Second Edition. Parallel process multigroup latent class growth analysis extracted sex-specific trajectory classes. Neurodevelopmental traits of children at age 9, autistic traits, attention deficit hyperactivity disorder (ADHD) traits, and cognitive ability were examined for females and males in each identified class. A 4-class model demonstrated the best fit. Moreover, a 4-class model that allowed for differences in class probabilities and means of growth parameters between females and males provided a better fit than a model assuming no sex differences. In the communication domain, females scored higher than their male counterparts in all four classes. In the daily living skills and socialization domains, the two higher adaptive classes (Class 1: females, 18.6%; males, 17.8%; Class 2: females, 48.8%; males, 49.8%) had similar trajectories for males and females, whereas in the two lower adaptive behavior classes (Class 3: females, 27.5%; males, 29.4%; Class 4: females, 5.1%; males, 3.0%), females had higher adaptive scores than their male counterparts. In Class 4, females were more likely to have autistic and ADHD traits exceeding the cutoffs, while males were more likely to have below-average IQ. Different trajectories in females and males suggest that adaptive skills may require adjustment based on the sex of the child, when standardizing scores, in order to achieve better early detection of skill impairment.

## Introduction

Adaptive behavior is an individual's acquired social and practical skills for application in typical everyday situations (1). These involve functional use of verbal and non-verbal communication, daily living skills (e.g., being able to take care of one's own health and safety), and socialization skills (e.g., behaving in a socially acceptable manner) (2, 3). Moreover, adaptive skills increase in complexity with age and must be understood within a developmental context (4).

Impairment of adaptive behaviors, especially socialization skills, has often been reported in individuals with autism spectrum disorders (ASD). Since adaptive behaviors predict functional outcomes of children with ASD, independent of symptoms, it is crucial to understand the developmental trajectories of adaptive behaviors. Thus, previous studies have focused on individuals with ASD or with an elevated likelihood of receiving a diagnosis of ASD (5–8). However, first, it is important to understand what trajectory patterns exist in the general population, and then, to determine which trajectory patterns children with ASD most often assigned to. In addition, adaptive behavior is an important developmental indicator for children with broader neurodevelopmental conditions (9). For example, individuals with attention deficit hyperactivity disorder (ADHD) and intellectual disability may experience challenges with adaptive behaviors (10). Therefore, it would be meaningful to explore the class structure in a general population including children with various neurodevelopmental conditions as well as children with typical development (TD). Moreover, it is meaningful to determine to which classes individuals with diverse neurodevelopmental traits would likely be assigned based on given class patterns.

Most crucially, to the best of our knowledge, there are no studies that have longitudinally investigated sex differences in adaptive behaviors, despite the reported sex differences in early milestone acquisition. For example, females are generally reported to achieve developmental milestones, including language acquisition and social–emotional development, earlier than males (11). In addition, the overall prevalence of neurodevelopmental disorders such as ASD and ADHD has been reported to be higher in males than in females (12). However, it is not known whether the class structure reported in previous studies differs between females and males, nor to which classes are females and males with neurodevelopmental traits assigned. Notably, recent studies have highlighted that the male-to-female ratio of prevalence is not as large as previously estimated, possibly because some ASD and ADHD cases in females may be overlooked (13, 14). Females are less likely to manifest distinct early signs of such disorders and are diagnosed at a later age than males (15). The bias toward greater risks for males than females may be attributed to previous studies conducted with predominantly male participants (16). A recent study also reported that females display specific neurodevelopmental phenotypes that are qualitatively different from those of males (17), thereby requiring same-sex comparison when assessing their early signs. It is crucial to examine whether there are sex-specific trajectories of adaptive behaviors in early developmental stages.

This study has two main objectives. First, we explored the trajectory classes of adaptive behaviors in a representative sample of children enrolled in and followed through our birth cohort study, using a latent class growth analysis. Second, this study aimed to evaluate sex differences in these trajectories and neurodevelopmental traits of children assigned to each trajectory class. We evaluated these trajectories while considering the effect of sex, assuming that heterogeneity by sex exists. Using a multigroup approach, a model including all children was compared to a model that allows for different class probabilities for females and males, and to another model that allows for different means of growth parameters (i.e., different trajectories) for females and males in addition to different class probabilities. We hypothesized that there would be greater variation in the classes identified in this study compared to studies using a sample of children with ASD or with an elevated likelihood for the condition. We also hypothesized that class membership and growth trajectories would differ between females and males, and that females would generally have higher adaptive behaviors than males from early childhood to school age, especially in communication and socialization domains. Children (both females and males) with higher neurodevelopmental traits than their same-sex peers would likely be assigned to the lower adaptive behavior classes.

## Methods

### Study Design and Participants

This study was conducted as part of an ongoing prospective cohort study, the Hamamatsu Birth Cohort Study for Mothers and Children (HBC Study), comprising mothers (*n* = 1,138) and their children (*n* = 1,258) (18, 19). The HBC Study invited all women who were in the first or second trimester of pregnancy who visited the Hospital of Hamamatsu University School of Medicine or the Kato Maternity Clinic between November 2007 and March 2011. Most (99%) of the enrolled mothers were Japanese. Adaptive behaviors were assessed when the children were 2.7, 3.5, 4.5, 6, and 9 years old. The 264 participants without any measures of adaptive behavior in the five measurements at any time point were excluded from the analysis, leaving 994 children and 893 mothers included in the analyses. The participating children are representative of the Japanese population in terms of demographic characteristics and standardized test scores (Supplementary Table 1). Further details of the study have been described previously (19).

All procedures contributing to this work comply with the ethical standards of the relevant national and institutional committees on human experimentation and with the Helsinki Declaration of 1975, as revised in 2008. All procedures were approved by the Institutional Review Board of the Hamamatsu University School of Medicine (Ref. 18-166, 19-9, 20-82, 22-29, 24-67, 24-237, 25-143, 25-283, E14-062, E14-062-1, E14-062-3, 17-037, 17-037-3, 20-233). Written informed consent was obtained from all caregivers for their own and their children's participation.

### Measures

#### Adaptive Behaviors

Daily functional abilities from early childhood to school ages were quantified using the Japanese version of the Vineland Adaptive Behavior Scales–Second Edition (VABS-II) (20, 21). The VABS-II is based on a semi-structured parental interview comprising four domains: communication, daily living skills, socialization, and motor skills. We used age-adjusted standard scores (mean = 100, SD = 15) for the three domains of communication, daily living, and socialization. Higher scores indicate better adaptive behaviors.

#### Neurodevelopmental Traits at 9 Years of Age

Autistic traits were evaluated using the Social Responsiveness Scale, Second Edition (SRS-2) school age form (22) comprising 65 items. The SRS-2 raw scores were converted to total T-scores (mean = 50, SD = 10), which were normalized based on a nationally representative standardization sample stratified by sex (22, 23). The translated version of the SRS-2 has been explored for validity in the general population, and high correlation (ICC = 0.66) with the Autism Diagnostic Interview-Revised (ADI-R), which is a research standard for establishing a diagnosis of autism, was confirmed. When used for primary screening of the general population, the optimal cutoff point was 53.5 for males (equivalent to T-score 60; sensitivity 0.91, specificity 0.48) and 52.5 for females (equivalent to T-score 62; sensitivity 0.89, specificity 0.41) (23). Accordingly, children with scores exceeding these cutoffs were considered to have autistic traits.

ADHD traits were evaluated using the Japanese version of the ADHD-Rating Scale (ADHD-RS) consisting of 18 items (24). The ADHD-RS has been shown to have appropriate psychometric properties for use as a screening, diagnostic, and treatment outcome measure (25). The subscales have also been found to have high internal consistency reliability, interrater reliability, discriminant validity, and significant correlations with other scales widely used in the assessment of ADHD in a representative sample of Japanese children. The percentile scores stratified by sex were obtained (25), and children with scores above the 85th percentile were classified as having ADHD traits.

Cognitive ability was assessed using the Japanese version of the Wechsler Intelligence Scale for Children-Fourth Edition (WISC-IV) (26), and full-scale IQ (mean = 100, SD = 15) was evaluated. Full-scale IQs below 85 was classified as below-average.

## Statistical Analysis

Using multigroup parallel process latent class growth analysis (27, 28), trajectories of adaptive behaviors were estimated in females and males. Three domains of adaptive behaviors, including communication, daily living skills, and socialization, were processed in parallel. In the first step, a single group latent class growth model was estimated using the entire sample. Growth parameters included the intercept (I), slope (S), and quadratic term (Q) for each of the three adaptive behaviors. Participants were assigned to the latent classes based on the most likely posterior probabilities (maximum-probability assignment rule). To achieve an appropriate number of classes, a sequence of models was fitted, and the optimum model was determined based on the following model fit indices: smallest Bayesian information criterion (BIC), sample size adjusted BIC, and Akaike's information criterion (AIC); *p* < 0.05 on the Lo–Mendell–Rubin likelihood ratio test (LMR-LRT) and the bootstrap likelihood ratio test (BLRT), and entropy (29–31). Theoretical justification and interpretability were also considered. In the next step, the following three types of multigroup latent class growth models were estimated: total invariance, partial invariance, and non-invariance models. In the total invariance model, both class probabilities and means of growth parameters (I, S, and Q) were constrained to be equal between females and males. The partial invariance model allows differences in class probabilities between females and males, but the means were constrained to be equal. The non-invariance model allows for differences in class probabilities and means between females and males. These models were compared by difference testing using log-likelihoods. Missing values were observed in 2.4% of the total data on adaptive behavior. The number of missing values was not associated with the scores of adaptive behaviors at each time point and with sex. Therefore, we employed the full information maximum likelihood algorithm under the assumption of missing at random (32). These analyses were conducted using Mplus 8.5 (33).

To examine the sex differences in neurodevelopmental traits at age 9 in each latent class, simple linear regression analysis was conducted. Since the test was repeated 12 times, *q*-values, which are the adjusted *p*-values using a false discovery rate (FDR) approach (34), were obtained. In addition, in order to compare the test results using different sample sizes, the effect sizes of the differences (η^2^) were calculated. The following benchmarks of the effect size provided by Cohen (35) were used: medium (η^2^ ≥ 0.06) and large (η^2^ ≥ 0.14). Raw ADHD-RS scores were logarithmically transformed because of non-normality. Sex differences in the number of children who exceeded the cutoff values were also evaluated using the chi-square test for each latent class, and the effect sizes of Cramér's V were calculated (36). These analyses were conducted using Stata 15.0 (37).

## Results

To detect the appropriate number of classes in a single group latent class growth model, we ran the model from one- to five-class solutions. The values of AIC, BIC, and adjusted BIC continued to decrease, but the rate of decrease from the 4-class to the 5-class solution was very small (Table 1). The *p*-values of BLRT were <0.001 up to the five-class solution. The *p*-values of the LMR-LRT were <0.05, up to the 4-class solution. The entropy values were sufficiently high for all class solutions. Based on the results of the LMR-LRT and theoretical justification, we determined that the 4-class solution was optimal.

**Table 1:** Fit indices of each class solution in the multigroup latent class growth model.

**Number of classes**	**1**	**2**	**3**	**4**	**5**
AIC	105,232.76	101,843.19	100,790.09	100,220.01	100,047.17
BIC	105,360.21	102,024.56	101,025.37	100,509.21	100,390.3
Adjusted BIC	105,277.63	101,907.05	100,872.92	100,321.82	100,167.97
Adjusted LMR-LRT *p*-value	–	<0.001	0.036	0.017	0.929
BLRT *p*-value	–	<0.001	<0.001	<0.001	<0.001
Entropy	–	0.886	0.848	0.865	0.823

The comparison of the multigroup latent class growth models revealed that the partial invariance model was better than the total invariance model [χ^2^(3) = 1,115.2, *p* < 0.001], and the non-invariance model was better than the partial invariance model [χ^2^(18) = 76.1, *p* < 0.001]. We therefore adopted the non-invariance model, in which the class probabilities and means were assumed to differ between females and males.

**Figure 1:**
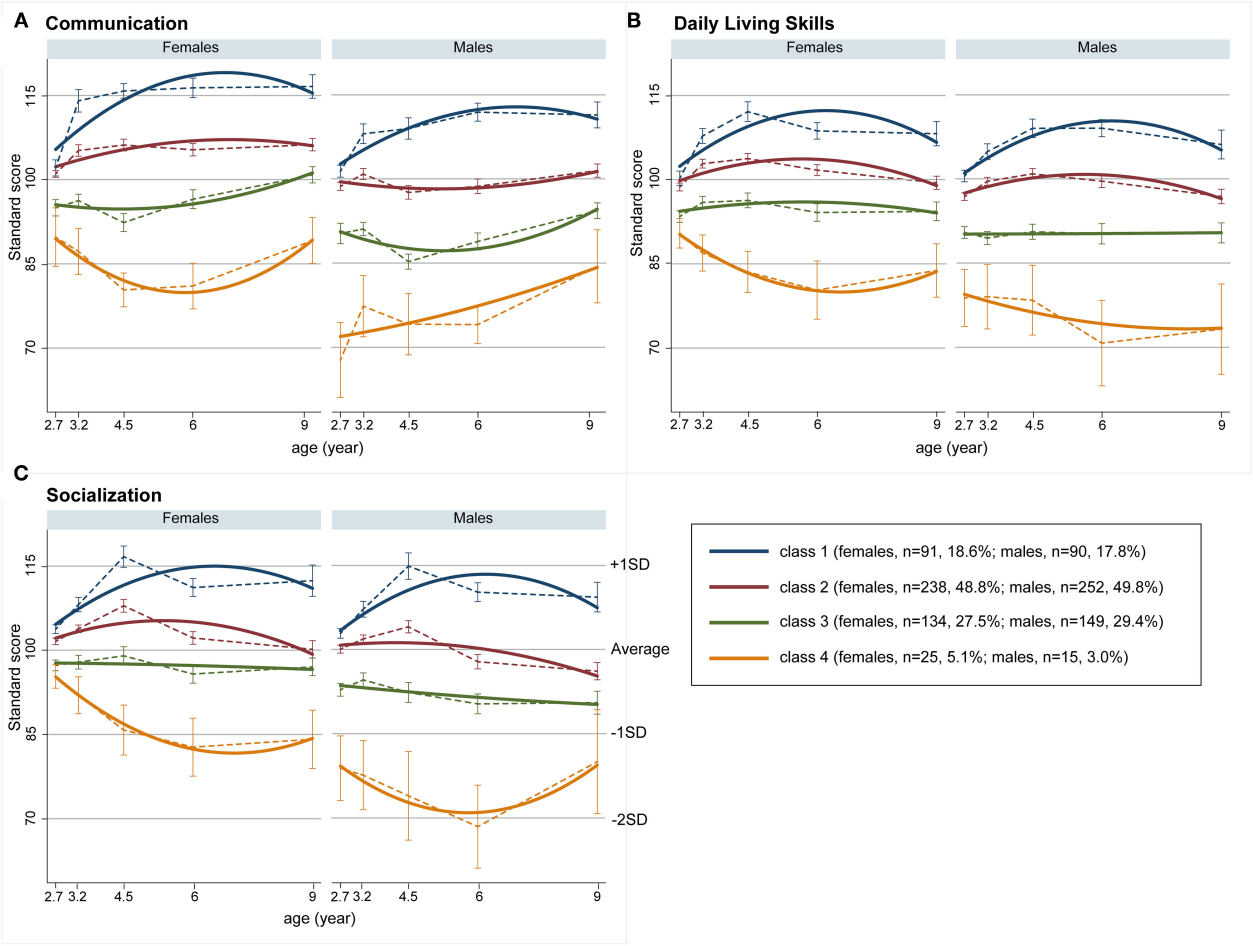
Growth trajectories of adaptive behaviors. **(A)** Communication, **(B)** daily living skills, and **(C)** socialization; bold lines represent estimated trajectories and dashed lines represent observed trajectories; error bars represent standard errors.

Table 2 shows estimated growth parameters in each sex and domain. Figure 1 shows estimated trajectories (bold lines) and observed values at each time-point (dashed lines with standard errors) in each latent class. Class 1 (females, 18.6%; males, 17.8%) was characterized with higher scores than the average (standard score of 100) in both sexes in all three domains during the follow-up period. Class 1 displayed a positive slope and negative quadratic term values in all domains (Table 2), with gentle inverse U-shape trajectories (Figure 1). The largest percentages of children were assigned to Class 2 (females, 48.8%; males, 49.8%). In the communication domain, females in Class 2 had moderately high scores and males had average scores, while in daily living skills and socialization domains, females and males exhibited similar trajectories (Figure 1). Class 3 (females, 27.5%; males, 29.4%) was characterized by below-average trajectories. Females assigned to Class 3 achieved scores close to average, whereas males had moderately low scores. Both females and males in this class obtained lower initial scores at 2.7 years of age than their peers in Class 2 in all three domains (Figure 1). Class 4 (females, 5.1%; males, 3.0%) had low scores in females and particularly in males. Females in this class had lower initial scores than those in Class 2 and displayed gentle *U*-shape trajectories (Figure 1). Males had low initial scores and relatively stable trajectories because slope parameters were not significant in all domains (Table 2).

**Table 2:** Estimated growth parameters in the multigroup latent class growth model.

**Latent classes**	**Class 1**		**Class 2**		**Class 3**		**Class 4**	
**Growth**	**Females**	**Males**	**Females**	**Males**	**Females**	**Males**	**Females**	**Males**
**parameters**	**(*n* = 91, 18.6%)**	**(*n* = 90, 17.8%)**	**(*n* = 238, 48.8%)**	**(*n* = 252, 49.8%)**	**(*n* = 134, 27.5%)**	**(*n* = 149, 29.4%)**	**(*n* = 25, 5.1%)**	**(*n* = 15, 3.0%)**
**Communication**
I	105.4^*^	102.8^*^	102.3^*^	99.6^*^	95.5^*^	90.7^*^	89.4^*^	72.0^*^
S	6.7^*^	4.8^*^	2.3^*^	−1.1	−1.0	−2.7^*^	−6.2^*^	1.3
Q	−0.8^*^	−0.6^*^	−0.3^*^	0.2	0.3^*^	0.6^*^	0.99^*^	0.12
**Daily living skills**
I	102.4^*^	101.2^*^	99.8^*^	97.6^*^	94.3^*^	90.3^*^	90.2^*^	79.5^*^
S	5.6^*^	5.3^*^	2.6^*^	2.3^*^	1.1^*^	0.02	−5.3^*^	−2.3
Q	−0.8^*^	−0.7^*^	−0.5^*^	−0.4^*^	−0.2^*^	0.004	0.7^*^	0.2
**Socialization**
I	104.6^*^	103.5^*^	102.2^*^	100.9^*^	97.7^*^	93.7^*^	95.3^*^	79.3^*^
S	5.4^*^	5.8^*^	2.4^*^	0.7	−0.08	−0.8	−6.3^*^	−5.4
Q	−0.7^*^	−0.8^*^	−0.5^*^	−0.3^*^	−0.02	0.03	0.7^*^	0.9^*^

* *p < 0.05. I, intercept; S, slope; Q, quadratic term*.

Table 3 summarizes sex differences in neurodevelopmental traits at age 9 in each latent class. The distribution of these neurodevelopmental traits are shown in Supplementary Figures S1–S4. Males in Class 2 had higher ADHD-RS total raw scores than females, with medium effect size. Table 4 shows the number of children who exceeded the cutoff values in each latent class. Sex differences in autistic traits were identified in Class 3 and Class 4. The number of children with autistic traits (SRS-2 T-score ≥60 for male and ≥62 for female) was higher for males than females in Class 3, whereas it was higher for females than males in Class 4 (Table 4). Sex differences in ADHD traits were identified in Class 2, Class 3, and Class 4. The number of children who exceeded the cutoff value for ADHD-RS (>85th percentile) was higher in males than females in Class 2 and 3, whereas it was the opposite in Class 4. Sex differences in cognitive ability were identified in Class 4, in which the number of children with below-average IQ was higher for males than females.

**Table 3:** Sex differences in neurodevelopmental traits at age 9 in each latent class.

		**Class 1**	**Class 2**	**Class 3**	**Class 4**
		**(female: *n* = 79;**	**(female: *n* = 198;**	**(female: *n* = 112;**	**(female: *n* = 21;**
		**male: *n* = 72)**	**male: *n* = 217)**	**male: n=131)**	**male: *n* = 10)**
SRS-2 total raw score; mean (SD)	Female	20.9 (11.1)	30.1 (14.5)	36.1 (17.1)	54.8 (27.0)
	Male	24.5 (12.5)	36.4 (16.0)	45.2 (20.5)	51.2 (21.1)
	Difference testing	β = 0.15, q = 0.12, η^2^ = 0.03	β = 0.20, q <0.001, η^2^ = 0.04	β = 0.23, q <0.001, η^2^ = 0.05	β = −0.07, q = 0.77, η^2^ = 0.005
ADHD-RS total score; mean (SD)	Female	3.2 (3.5)	4.4 (4.7)	5.5 (5.7)	11.5 (7.5)
	Male	4.4 (4.9)	8.4 (7.8)	10.8 (9.1)	12.0 (7.9)
	Difference testing	β = 0.15, q = 0.12, η^2^ = 0.008	β = 0.30, q <0.001, η^2^ = 0.08^*^	β = 0.32, q <0.001, η^2^ = 0.05	β = 0.03, q = 0.86, η^2^ = 0.01
WISC-IV full scale IQ; mean (SD)	Female	108.4 (11.1)	105.2 (12.7)	98.4 (11.6)	83.6 (16.4)
	Male	109.7 (14.5)	100.8 (12.8)	96.0 (13.5)	78.4 (15.0)
	Difference testing	β = 0.05, q = 0.66, η^2^ = 0.002	β = −0.17, q = 0.002, η^2^ = 0.03	β = −0.10, q = 0.21, η^2^ = 0.01	β = −0.15, q = 0.59, η^2^ = 0.02

* *η^2^ ≥ 0.06 (medium effect size). SRS-2, the Social Responsiveness Scale, Second Edition; ADHD-RS, ADHD-Rating Scale; WISC-IV, the Wechsler Intelligence Scale for Children-Fourth Edition Compared to the number of children assigned in the latent class growth analysis, there were attrition of 13% for females and 20% for males in Class 1, 17% for females and 14% for males in Class 2, 16% for females and 12% for males in Class 3, and 16% for females and 33% for males in Class 4*.

**Table 4:** Sex differences in the number of children who exceeded the cut-off values of neurodevelopmental traits at age 9 in each latent class.

		**Class 1**	**Class 2**	**Class 3**	**Class 4**
		**(female: *n* = 79;**	**(female: *n* = 198;**	**(female: *n* = 112;**	**(female: *n* = 21;**
		**male: *n* = 72)**	**male: *n* = 217)**	**male: n=131)**	**male: *n* = 10)**
Autistic traits (SRS-2 total T-score ≥60 for male and ≥62 for female); *n* (%)	Female	1 (1.3)	19 (9.6)	22 (19.6)	11 (52.4)
	Male	2 (2.8)	31 (14.3)	41 (31.3)	2 (20.0)
	Difference testing	χ^2^(1) = 0.44, q = 0.57, V = 0.05	χ^2^(1) = 2.1, q = 0.28, V = 0.07	χ^2^(1) = 4.3, q = 0.20, V = 0.13^†^	χ^2^(1) = 2.9, q = 0.21, V = −0.31^‡^
ADHD traits (ADHD-RS score >85th percentile); *n* (%)	Female	3 (3.8)	22 (11.1)	20 (17.9)	11 (52.4)
	Male	4 (5.6)	39 (18.0)	46 (35.1)	8 (40.0)
	Difference testing	χ^2^(1) = 0.26, q = 0.66, V = 0.04	χ^2^(1) = 3.9, q = 0.21, V = 0.10^†^	χ^2^(1) = 9.1, q = 0.04, V = 0.19^†^	χ^2^(1) = 0.42 q = 0.62, V = −0.12^†^
Below-average IQ (WISC-IV full scale IQ <85); *n* (%)	Female	1 (1.3)	8 (4.1)	11 (9.9)	9 (42.9)
	Male	0 (0)	18 (8.3)	21 (16.0)	5 (62.5)
	Difference testing	χ^2^(1) = 0.92, q = 0.46, V = −0.08	χ^2^(1) = 3.15, q = 0.23, V = 0.09	χ^2^(1) = 1.96, q = 0.32, V = 0.09	χ^2^(1) = 0.90, q = 0.46, V = 0.18^†^

† *Cramér's V ≥ 0.10 (weak association).*

‡ *Cramér's V ≥ 0.20 (moderate association).*

*SRS-2, the Social Responsiveness Scale, Second Edition; ADHD-RS, ADHD-Rating Scale; WISC-IV, the Wechsler Intelligence Scale for Children-Fourth Edition Compared to the number of children assigned in the latent class growth analysis, there were attrition of 13% for females and 20% for males in Class 1, 17% for females and 14% for males in Class 2, 16% for females and 12% for males in Class 3, and 16% for females and 33% for males in Class 4*.

## Discussion

The present study identified four distinct trajectories of adaptive behaviors from early childhood to school age in a general population. Although the number of identified classes was greater than in previous studies (5–8), trajectories were relatively stable, which is similar to the existing literature. However, we identified, for the first time, that class assignment and estimated trajectories differed between females and males. In the communication domain, females scored higher than their male counterparts in all four classes throughout the observation period. This was not the case in the daily living skills and socialization domains; the two higher adaptive classes had similar trajectories for males and females, but females assigned to the lower two classes had higher adaptive scores than their male counterparts. The results also showed that females assigned to the class with the lowest adaptive skills (Class 4) already had lower scores than their same-sex peers at around the age of 3 years, and then, they exhibited declining trajectories compared with females assigned to the other three classes. These results suggest that comparison with same-sex peers is required for early detection of impairment in adaptive skills, especially in females, and that sex-specific standard scores for adaptive behaviors are necessary.

For each domain of adaptive behaviors, females scored higher than males in communication in all four classes during the follow-up period. In children with TD, it has been reported that females acquire language earlier than males (11), but evidence is limited (38), and little is known about sex differences in communicative adaptive behaviors. In the communication domain, females with TD exhibited significantly better adaptive skills compared to males with TD (39, 40), which is consistent with our findings. Females with TD were also reported to possess higher scores than males with TD in daily living skills, but scores did not differ between females and males in socialization (40). In contrast, in daily living skills and socialization domains, trajectories in two higher adaptive behavior classes were similar for females and males in the present study. The previous studies reported that males and females with ASD exhibited no significant differences in adaptive behaviors (39, 40). Conversely, in the two lower classes in this study, males generally scored lower than females. The results are not comparable because previous studies included children who had already been diagnosed with ASD, whereas the present study included children with various developmental conditions. Further study is needed on sex differences in adaptive behavior trajectories for children with a variety of conditions as well as children with TD.

The relationship between class assignment and neurodevelopmental traits at age 9 differed between females and males. Males assigned to Class 3 were more likely to have autistic and ADHD traits exceeding the cutoff values, whereas females assigned to Class 4 were more likely to have autistic and ADHD traits. These neurodevelopmental traits were reported to cause a decline in adaptive behaviors (41–43). Females in Class 4 (52.4% having autistic and 52.4% having ADHD traits above sex-stratified cutoffs) already had lower adaptive scores before age 3 compared to their same-sex peers, and this gap widened as time progressed. In addition, their adaptive scores were lower than males in Class 3. A cross-sectional study examining sex differences in adaptive behaviors also found that females with ASD showed lower adaptive functions than males with ASD at older ages, despite females performing better at younger ages (44), which is consistent with our results. These results imply that some females may not be diagnosed with ASD in early childhood and miss opportunities to receive early interventions, leading to the failure to acquire age-appropriate adaptive behaviors over time. Therefore, our results highlight the importance of early interventions to prevent further declines in adaptive behaviors in females with these challenges and those with elevated neurodevelopmental traits. Males assigned to Class 4 displayed lower cognitive scores. Although the percentages exceeding the cutoff for autistic and ADHD traits were not large, the raw scores for those traits were high, suggesting that some males with severe traits were included in Class 4. Males assigned to Class 4 also had demographic characteristics such as lower birth weight, higher birth order, and higher parental age at birth (Supplementary Table 2). In addition to the neurodevelopmental traits of children, these characteristics may have influenced their adaptive behaviors. Males with ADHD traits exceeding the cutoffs were assigned to Class 3 as well as Class 4 (Table 4). Males assigned to Class 3 also already had lower adaptive scores before age 3 compared to their same-sex peers, but their adaptive behaviors did not decline with age. In addition, females and males assigned to Class 4 showed slight improvements in some adaptive domains at age 9. The reason for these findings is unclear, but one possibility is that adaptive functions were retained or improved in some children by therapeutic interventions such as speech and behavioral therapy, and/or specific educational strategies. To make early interventions possible, it is necessary to identify early signs and patterns of declining adaptive behaviors. Such early signs could be less likely to be identified in females than in males when children of the same age group are taken as a whole, suggesting that it is important to compare the data of individuals against same-sex peers. As suggested in the field of ASD in assessing social functioning (45), establishing sex-specific standard scores would be a useful index for comparison with same-sex peers in the assessment of adaptive behaviors. Because adaptive behaviors are acquired, thus, modifiable, the expansion of the evaluation system and the early support and intervention systems for promoting adaptive behaviors are increasingly critical for effective home, family, school, community, and vocational planning throughout life (46).

## Strength and Limitations

In the present study, we longitudinally examined sex differences in the trajectories of adaptive behaviors from early childhood to school age using a representative sample and advanced statistical methodologies. However, there are some limitations that must be considered when interpreting the findings. We excluded 264 children from the analysis because they had no measurements on adaptive behaviors. A comparison of the demographic characteristics of the groups included and excluded from the analysis revealed no differences regarding birth weight or gestational age of the children; however, parental age at birth differed between groups. Parental age has been associated with neurodevelopmental disorders (47) and could have influenced the results of the present study. Another limitation is the relatively low prevalence, because our cohort was a representative sample from the general population. Therefore, the lowest adaptive behavior class did not possess sufficient statistical power for subsequent analysis. Future studies involving larger cohorts are required to confirm these results. Finally, neurodevelopmental traits examined in the present study were based on the cutoff point of the scales, but not diagnosis. Neurodevelopmental traits above a cutoff value do not necessarily correspond to a clinical diagnosis. Further research is needed to determine the trajectory of adaptive behavior of children with the actual diagnosis in the general population.

## Data Availability Statement

The data analyzed in this study is subject to the following licenses/restrictions: The data that support the findings of this study are available upon request from the corresponding author. The data are not publicly available because they contain information that could compromise the privacy of research participants. Requests to access these datasets should be directed to KT, tsuchiya@hama-med.ac.jp.

## Ethics Statement

The studies involving human participants were reviewed and approved by the Institutional Review Board of the Hamamatsu University School of Medicine. Written informed consent to participate in this study was provided by the participants' legal guardian/next of kin.

## Author Contributions

TN conceptualized the study, conducted the statistical analysis, and drafted the original manuscript. TK, THi, MT, and MA provided major revisions to the drafts. AS and KT supervised and critically revised the manuscript. AO, THa, TI, MR, HK, ST, YN, and NT provided feedback and edited the final manuscript. TN, AO, THa, TI, and KT conducted the data acquisition. All authors contributed to the manuscript and approved the submitted version.

## Funding

This work was supported by the Japan Society for the Promotion of Science, Grants-in-Aid for Scientific Research (grant numbers 19H03582 to KT and 20K07941 to TN), and by AMED (grant number JP21gk0110039h0003 to KT).

## Conflict of Interest

The authors declare that the research was conducted in the absence of any commercial or financial relationships that could be construed as a potential conflict of interest.

## Publisher's Note

All claims expressed in this article are solely those of the authors and do not necessarily represent those of their affiliated organizations, or those of the publisher, the editors and the reviewers. Any product that may be evaluated in this article, or claim that may be made by its manufacturer, is not guaranteed or endorsed by the publisher.
