# Supplementary material for: Trajectories of Adaptive Behaviors During Childhood in Females and Males in the General Population
Source: Front Psychiatry. 2022 Mar 23;13:817383. doi: 10.3389/fpsyt.2022.817383 (PMC8983934; doi:10.3389/fpsyt.2022.817383)
Supplement: Supplementary file 1 [file Data_Sheet_1.pdf]

Supplementary Material

Supplementary Table 1. Comparison of demographic characteristics between participants in HBC Study and national statistics of Japan

	HBC Study participants	National statistics of Japan[1]
Child characteristics (n = 1258)		
Male sex; n (%)	648 (52%)	527,657 (51%)
Birthweight (g); mean (SD)	2942 (435)	3000
Low birthweight; n (%)	160 (12.7%)	98,624 (10%)
Gestational age (week); mean (SD)	38.9 (1.6)	Data not available
Preterm birth; n (%)	82 (6.5%)	59,235 (5.8%)
Parity (primipara); n (%)	626 (50%)	481,418 (47%)
Parity (multipara); n (%)	632 (50%)	547,858 (53%)
Twins; n (%)	38 (3%)	19,901 (2%)
SRS-2 total raw score; mean (SD)	34.3 (18.1)	32.5 (18.2)[2]
SRS-2 total T-score; mean (SD)	50.9 (9.9)	50.0 (10.0)
WISC-IV full scale IQ; mean (SD)	101.6 (14.0)	100.0 (15.0)
Parental characteristics (n = 1138)		
Mother's age at birth (y); mean (SD)	31.4 (5.1)	31.2
Father's age at birth (y); mean (SD)	33.3 (5.9)	33.2
Mother's educational year (y); mean (SD)	13.8 (2.0)	Data not available
Father's educational year (y); mean (SD)	14.1 (2.7)	Data not available
Annual household income (million JPY); mean (SD)	6.09 (2.82)	6.28

Abbreviations: HBC Study, Hamamatsu Birth Cohort Study for Mothers and Children; SRS-2, Social Responsiveness Scale, Second Edition; WISC-IV, Wechsler Intelligence Scale for Children-Fourth Edition

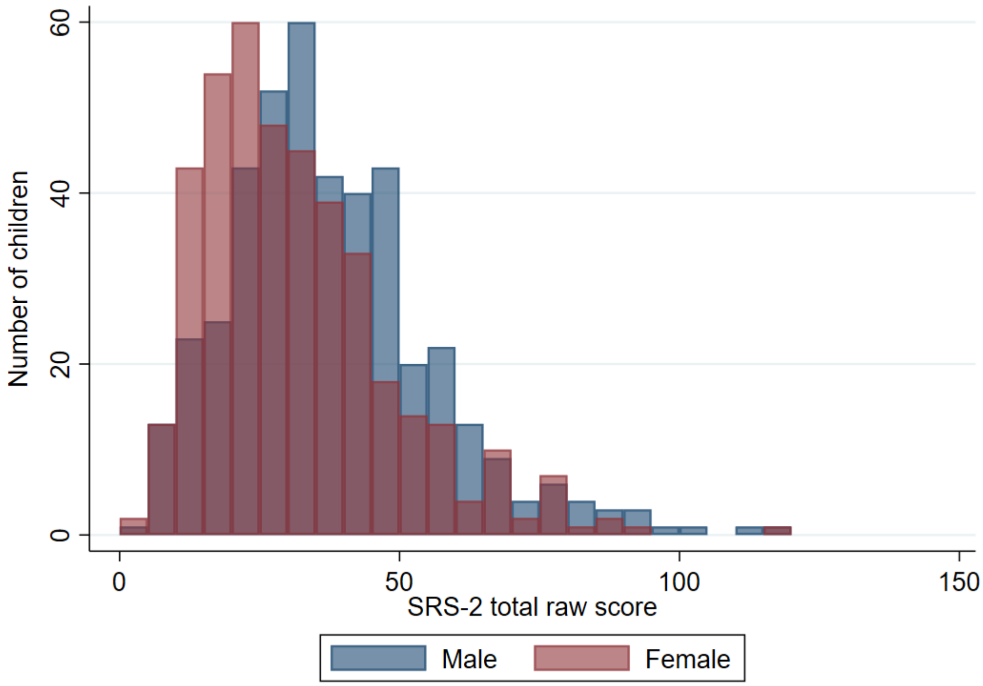

Figure S1. Distribution of Social Responsiveness Scale, Second Edition (SRS-2) total raw scores

Regarding the reliability and validity of the SRS-2 in our sample, Cronbach's α was 0.92, and the correlation coefficient between total scores of SRS-2 and total difficulty scores of the strength and difficulties questionnaire (SDQ)[3] was 0.68.

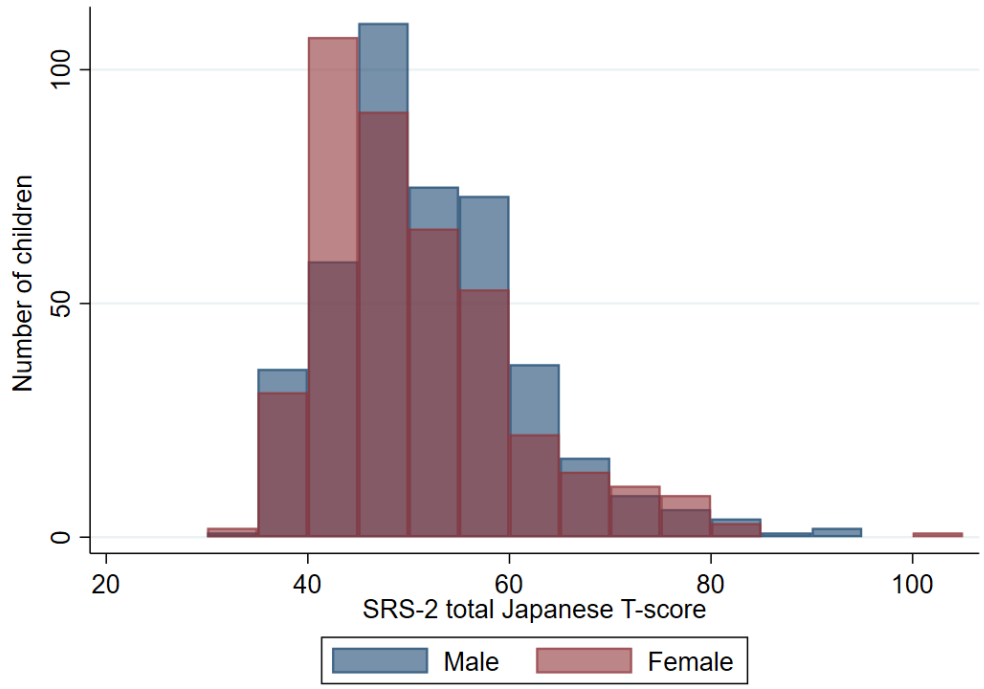

Figure S2. Distribution of Social Responsiveness Scale, Second Edition (SRS-2) total T-scores

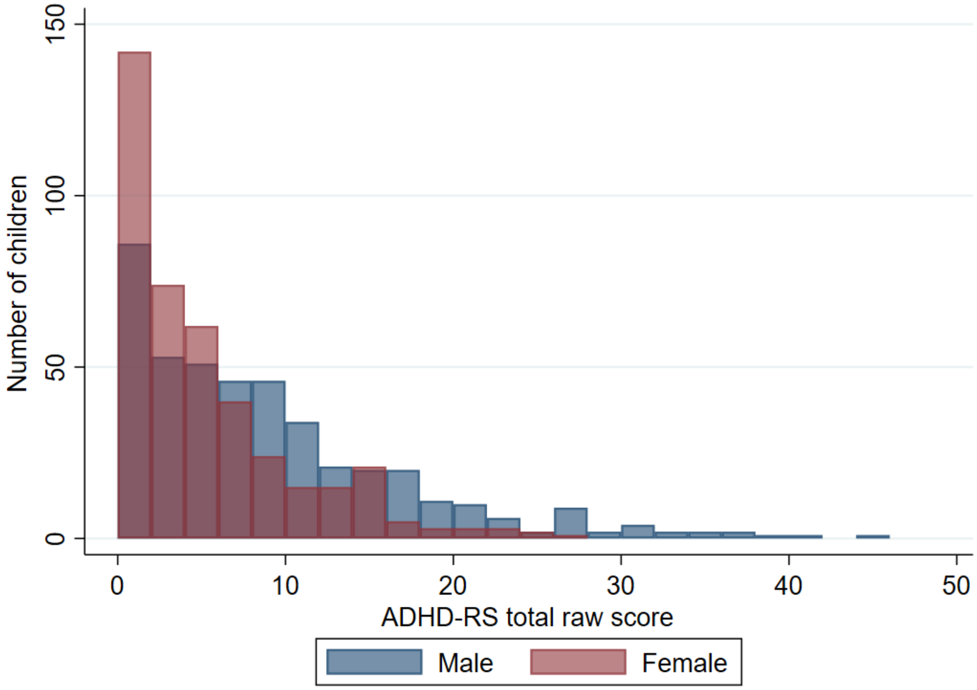

Figure S3. Distribution of ADHD-Rating Scale (ADHD-RS) total raw scores

Cronbach's α for the two subscales of ADHD-RS was 0.90 (hyperactive/impulsive) and 0.80 (inattentive). The correlation coefficient between the total scores of ADHD-RS and scores of the hyperactivity/inattention subscale of SDQ[3] was 0.69.

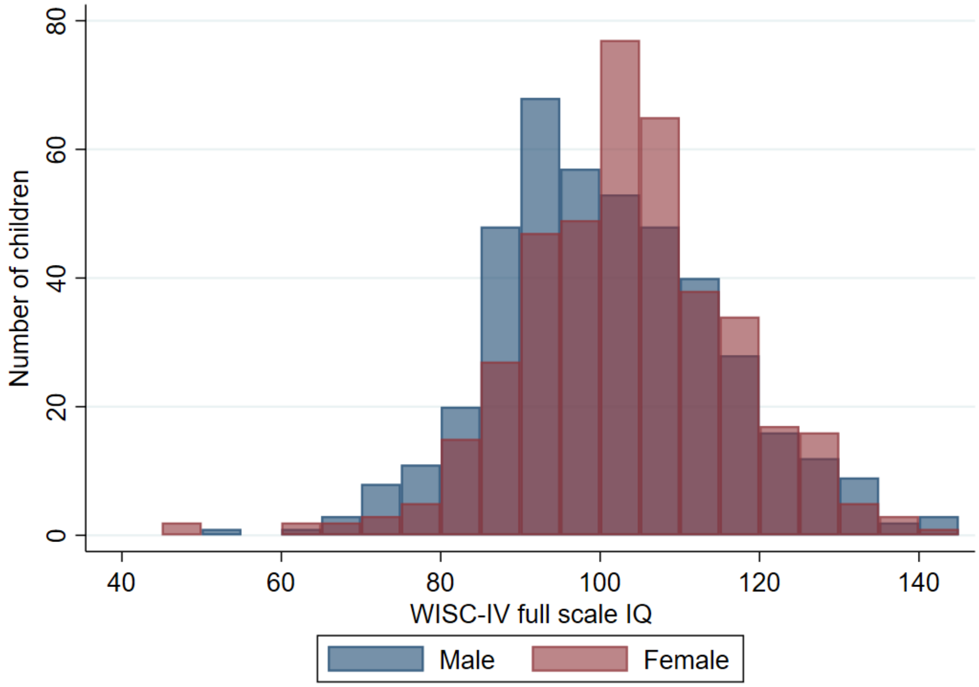

Figure S4. Distribution of the Wechsler Intelligence Scale for Children-Fourth Edition (WISC-IV) full-scale IQ

Supplementary Table 2. Demographic characteristics of children assigned to each latent class

	Class 1		Class 2		Class 3		Class 4	
	female (n=91)	male (n=90)	female (n=238)	male (n=252)	female (n=134)	male (n=149)	female (n=25)	male (n=15)
Birthweight (kg); mean (SD)	2.9 (0.4)	3.1 (0.4)	2.9 (0.4)	3.0 (0.5)	2.8 (0.5)	2.9 (0.5)	2.9 (0.4)	2.6 (0.6)
Gestational age (week); mean (SD)	38.9 (1.4)	39.1 (1.5)	39.1 (1.4)	38.9 (1.8)	38.9 (1.8)	38.8 (1.5)	38.7 (1.3)	38.0 (2.4)
Parity (primipara); n (%)	50 (55.0)	43 (47.8)	133 (55.9)	134 (53.2)	54 (40.3)	67 (45.0)	15 (60.0)	5 (33.3)
Parental income at birth (million JPY); mean (SD)	7.1 (3.1)	6.5 (3.2)	6.6 (3.1)	5.9 (2.7)	5.6 (2.2)	5.4 (2.0)	4.7 (1.9)	6.1 (2.3)
Mother's age at birth (year); mean (SD)	32.0 (5.2)	32.7 (4.4)	31.5 (4.8)	31.0 (5.3)	32.1 (5.1)	32.0 (4.9)	31.2 (6.0)	35.5 (3.8)
Father's age at birth (year); mean (SD)	33.8 (5.7)	33.9 (5.4)	33.4 (5.7)	32.5 (5.6)	34.4 (5.8)	33.7 (6.1)	34.2 (6.3)	35.8 (4.5)
Mother's education (year); mean (SD)	14.2 (2.0)	14.4 (1.8)	14.0 (2.1)	13.9 (1.9)	13.7 (1.9)	13.5 (1.7)	13.1 (1.3)	13.4 (1.9)
Father's education (year); mean (SD)	14.8 (2.8)	14.9 (2.8)	14.4 (2.7)	14.2 (2.7)	13.9 (2.4)	13.7 (2.3)	12.6 (2.2)	13.7 (3.0)

Reference

[1] Ministry of Health Labour and Welfare. Vital Statistics in Japan. 2018. https://www.mhlw.go.jp/english/database/db-hw/dl/81-1a2en.pdf [Accessed October 21, 2021]

[2] Kamio, Y., Inada, N., Moriwaki, A., Kuroda, M., Koyama, T., Tsujii, H., Kawakubo, Y., Kuwabara, H., Tsuchiya, K. J., Uno, Y. & Constantino, J. N. (2013). Quantitative autistic traits ascertained in a national survey of 22 529 Japanese schoolchildren. *Acta Psychiatr Scand*, 128:45-53.

[3] Goodman R. The Strengths and Difficulties Questionnaire: a research note. J Child Psychol Psychiatry (1997) 38:581-6. DOI: 10.1111/j.1469-7610.1997.tb01545.x
